# The Usual Presentation of an Unusual Case: Spontaneous Primary Splenic Cyst Rupture

**DOI:** 10.7759/cureus.25097

**Published:** 2022-05-18

**Authors:** Yucel Aydin, Bhavya Vemuri, Clifford Berg

**Affiliations:** 1 Internal Medicine, Saint Mary's Hospital, Waterbury, USA

**Keywords:** nonsurgical treatment, hemoperitoneum, spontaneous splenic cyst rupture, primary splenic cyst, acute abdomen

## Abstract

Acute abdominal pain is one of the most common reasons for emergency admissions. Even though initial differentials are wide, a physician is able to narrow them down with detailed history, careful physical examination, and appropriate laboratory tests along with imaging studies. Unfortunately, some of the cases do not have an established diagnosis despite multiple blood work and imaging studies in the emergency department. In such conditions, physicians' recognition of rare diseases generally avoids extra costs for additional investigations, unnecessary consultations, and most importantly wasting valuable time in life-threatening conditions in emergency settings.

Here, we report a 30-year-old woman with acute severe abdominal pain and hemodynamic instability who was found to have ascites that was actually hemoperitoneum secondary to spontaneous primary non-parasitic splenic cyst rupture. The primary splenic cyst is an extremely rare entity and is often found on imaging incidentally. A few case reports regarding primary splenic cyst and its complications were published in the literature. Since it is an exceptionally uncommon condition, there is no consensus on treatment. We aimed to increase the understanding of spontaneous primary splenic cyst rupture and its management among healthcare providers with this case report.

## Introduction

Acute abdomen refers to sudden onset, localized, or generalized severe pain in the abdomen necessitating urgent care. It could be due to many reasons including infection, inflammation, obstruction, perforation, or vascular occlusion. A thorough history along with a physical examination is generally enough for diagnosis. Laboratory tests and imaging studies are utilized to confirm the diagnosis by physicians. However, some cases remain undiagnosed even after extensive workup. In these cases, increased awareness by healthcare providers for rare diseases that cause acute abdomen saves critical time for patient care and decreases excess cost and unnecessary interventions. The non-parasitic splenic cyst is a rare condition that is classified into two main groups: primary and secondary [1]. While primary splenic cysts could be of congenital, neoplastic, or dermoid origin, secondary splenic cysts either originated from trauma or necrosis. The primary non-parasitic splenic cyst is often detected as an incidental finding in imaging studies in individuals; however, some patients present with localized left upper quadrant symptoms due to increased size or generalized symptoms due to complications including infection and rupture. The potential complications of infection or rupture are especially in the setting of trauma, which is the most common cause of splenic rupture in patients with a normal or cysted spleen. Spontaneous primary splenic cyst rupture without trauma is occasionally identified in the literature [2]. Moreover, the treatment of splenic cyst and its complications is still controversial due to the rarity of the disease. Here, we aimed to discuss a rare case of spontaneous primary splenic cyst rupture that was successfully managed with conservative treatment.

## Case presentation

**Figure 1:**
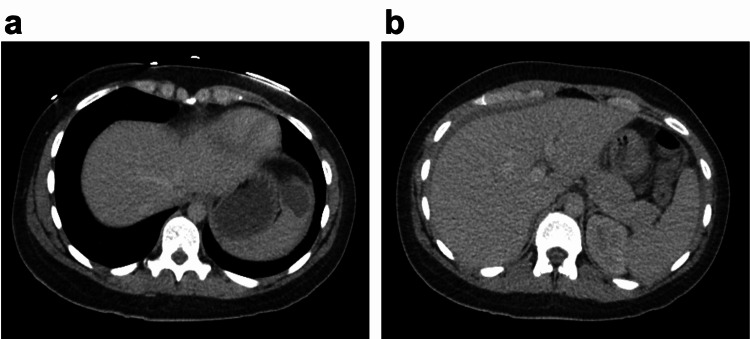
Axial CT images of the splenic cyst (a) and hemoperitoneum (b)

A 30-year-old woman presented to the emergency department with acute onset severe diffuse abdominal pain. She reported that she woke up early morning due to constant abdominal pain radiating to the back with 8/10 severity. She did not recall any trauma to the chest or abdomen. She was a sexually active, heterosexual woman using condoms and has regular menstrual cycles; the last one was three weeks ago. Her heart rate was 118/min, and the rest of her vital signs were normal. Her examination was also notable for moderate abdominal distension with no rebound or guarding, but her physical examination was otherwise normal. Laboratory data were significant for normocytic anemia with hemoglobin of 10.4 g/dL and neutrophilic leukocytosis of 19.8 x 10^9^/L. The serum pregnancy test was negative excluding the possibility of ectopic pregnancy. Computed tomography (CT) of the abdomen and pelvis with intravenous contrast revealed moderate ascites in the abdomen and pelvis and a splenic cyst of 2.5 cm x 2.5 cm (Figure 1, Panels a and b).

**Figure 2:**
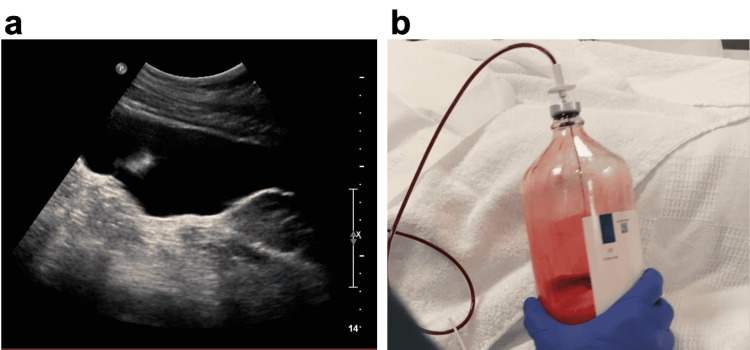
IR-guided paracentesis: an ultrasonographic image of ascites (a) and draining frank blood (b)

The patient was admitted to the medical floor overnight for further workup of ascites and severe abdominal pain. Repeat hemoglobin in next morning was 7.2 g/dL, but vitals remained stable other than persistent tachycardia. Interventional radiology-guided paracentesis yielded 500 mL of frank blood consistent with hemoperitoneum (Figure 2, Panels a and b). Retrospectively, ascites defined by CT of the abdomen was reevaluated by a radiologist, and the density of ascites was found to be 38 Hounsfield units (HU), which was similar to the density of blood in the aorta. The fluid analysis also confirmed hemoperitoneum with an RBC count of 8663 M/µL. Surgery was consulted immediately with the diagnosis of spontaneous rupture of the presumably primary splenic cyst. The surgery team considered conservative management in the critical care unit for close hemodynamic monitoring and serial abdominal exams along with frequent hemoglobin checks, given moderate symptoms. Interventional radiology-guided angiography to rule out aneurysms was deferred at this time because the splenic artery looks normal in the CT scan. The patient was discharged as hemoglobin did not drop any further and abdominal pain subsided during her stay. She did not report any active symptoms in a three-month follow-up upon discharge. The surgery team decided to follow up closely for recurrence with the plan of splenectomy if cyst rupture recurs.

## Discussion

Acute abdomen requires rapid detection of etiology and appropriate treatment and follow-up decisions in the emergency room. Even though most of the cases are straightforward for clinicians, some cases could be challenging in terms of diagnosis and management decisions. Splenic cysts are generally incidental findings on imaging modalities in people without any symptoms. The primary splenic cyst can cause symptoms either when it is large or when it is complicated by infection, rupture, bleeding, or hemoperitoneum. Splenic rupture is a life-threatening emergency that frequently occurs secondary to trauma. Non-traumatic rupture of the spleen is extremely uncommon and usually related to underlying pathological conditions including hematological diseases, neoplasms, inflammations, and infections.

Here, we present a non-traumatic splenic rupture in the setting of a primary splenic cyst that was successfully managed with conservative treatment. To date, less than 1000 individuals with splenic cysts and only 13 cases with ruptured splenic cysts have been reported in the literature [3-5]. The most common symptom of patients with splenic rupture is left upper quadrant abdominal pain that later generalizes with abdominal distention and rigidity. Pallor, tachycardia, hypotension, and oliguria are also expected as signs of bleeding. Paracentesis with the aspiration of fresh blood is useful to diagnose intraperitoneal hemorrhage. Point-of-care ultrasound (POCUS) or focus assessment with sonography in trauma (FAST) has been used at the bedside in emergency settings to address specific clinical questions and speed the diagnosis and treatment of patients. Over the last three decades, the use and application of ultrasound have expanded to include multiple diagnostic studies and procedural uses and become an integral part of emergency assessments. Our patient was a good candidate for POCUS and POCUS-guided paracentesis if splenic rupture was suspected within the initial hours of admission and would prevent diagnostic delay. Fortunately, our case did not suffer from the late diagnosis.

Splenic cysts are globally divided into two groups: parasitic cysts (secondary to *Echinococcus granulosus* infection) and non-parasitic cysts. Non-parasitic cysts can be subclassified into primary (congenital, neoplastic, and dermoid) and secondary cysts (trauma and necrosis). Most splenic cysts are acquired cysts in the setting of trauma that does not have an endothelial lining (pseudocyst) in contrast to congenital cysts. Radiologically, splenic cysts are fluid-density lesions. Ultrasound usually demonstrates an anechoic to hypoechoic well-defined intrasplenic lesion with no septations unless complicated. Splenic cysts typically are well-defined, fluid-attenuation, unilocular masses with imperceptible walls by CT. A CT is able to identify cyst wall calcifications and septations very well. Magnetic resonance (MR) shows splenic cysts as well-defined cystic non-enhancing lesions with low signal intensity on T1 and very high signal intensity on T2. MR is also useful for understanding the relationship between the cyst, the spleen, and the surrounding organs [6].

There has been no consensus on the treatment of splenic cysts due to a limited number of cases even though many distinctive approaches have been reported, including conservative treatment, spleen-saving procedures, and total splenectomy [7]. Total splenectomy is generally a choice of treatment in asymptomatic splenic cysts greater than 5 cm, any size of splenic cysts with symptoms and complicated cysts [6]. Due to the increasing awareness of the immunologic function of the spleen, spleen-saving techniques including percutaneous drainage, fenestration, marsupialization, and partial splenectomy or conservative management with close monitoring have more interest among surgeons, especially in hemodynamically stable patients instead of total splenectomy in order to prevent the need of vaccinations, risk of encapsulated organism infections, and prolonged antibiotic use following splenectomy [7].

Partial splenectomy saves more than 25% of parenchymal tissue, which is generally enough to conserve the immunologic function of the spleen without increasing the risk of relapse. Marsupialization or partial cystectomy is another option for splenic cysts that decrease surgery time with minimal risk of recurrence [6]. In our patient, the surgery team preferred to manage the patient conservatively with closer monitoring in the surgical ICU, given moderate abdominal symptoms and stable vitals other than tachycardia.

## Conclusions

Spontaneous splenic rupture due to a primary splenic cyst is an extremely rare condition, and clinicians should have a high index of suspicion for diagnosis, especially in patients that are presenting with an unexplained fluid in the abdomen with anemia or hemodynamic instability. In such cases, healthcare providers should be paying more attention to the spleen and its pathologies in particular. Delayed diagnosis and management of primary splenic cyst jeopardize patient's safety and can result in serious consequences including death.

## References

[1] Morgenstern L (2002). Nonparasitic splenic cysts: pathogenesis, classification, and treatment. J Am Coll Surg.

[2] Res LC, Knook MT, Hazelbag HM, Guicherit OR (2019). Spontaneous rupture of a non-parasitic splenic cyst. BMJ Case Rep.

[3] Doolas A, Nolte M, McDonald OG, Economou SG (1978). Splenic cysts. J Surg Oncol.

[4] Robbins FG, Yellin AE, Lingua RW, Craig JR, Turrill FL, Mikkelsen WP (1978). Splenic epidermoid cysts. Ann Surg.

[5] Inokuma T, Minami S, Suga K, Kusano Y, Chiba K, Furukawa M (2010). Spontaneously ruptured giant splenic cyst with elevated serum levels of CA 19-9, CA 125 and carcinoembryonic antigen. Case Rep Gastroenterol.

[6] Macheras A, Misiakos EP, Liakakos T, Mpistarakis D, Fotiadis C, Karatzas G (2005). Non-parasitic splenic cysts: a report of three cases. World J Gastroenterol.

[7] Hansen MB, Moller AC (2004). Splenic cysts. Surg Laparosc Endosc Percutan Tech.

